# Incidence and medical costs of lupus in Spanish hospitals: a retrospective database analysis

**DOI:** 10.1186/s13023-024-03077-1

**Published:** 2024-02-16

**Authors:** Josep Darba, Meritxell Ascanio, Ainoa Agüera

**Affiliations:** 1 Department of Economics, University of Barcelona, Diagonal 690, 08034 Barcelona, Spain; 2 BCN Health Economics & Outcomes Research S.L., Travessera de Gràcia, 62, 08006 Barcelona, Spain

**Keywords:** Systemic lupus erythematosus, SLE, Cutaneous lupus erythematosus: CLE, Incidence, Comorbidity, Spain

## Abstract

**Background:**

This study aimed to assess the comorbidity profile, use of healthcare resources and medical costs of patients with systemic lupus erythematosus (SLE) and cutaneous lupus erythematosus (CLE) treated at the hospital level in Spain.

**Methods:**

Admission records of patients with SLE and CLE that were registered between January 2016 and December 2020 were obtained from a Spanish hospital discharge database and analyzed in a retrospective multicenter study.

**Results:**

329 patients met the criteria; 64.44% were female and 35.56% were male, with a median age of 54.65 years. Mean Charlson comorbidity index (CCI) was 2.75 in the index admission. 31.61% of the patients suffered essential hypertension, 21.96% suffered asthma and 19.76% suffered hyperlipidemia. Mortality rate was 3.95%. The most common medical procedure was heart ultrasound (19.45%) and introduction in peripheral vein of anti-inflammatory with a percutaneous approach (17.93%). Mean admission cost was €6355.99.

**Conclusions:**

Lupus patients showed a higher incidence and prevalence in the female population, with associated cardiac diseases as the main secondary conditions.

## Background

Systemic lupus erythematosus (SLE) is a chronic autoimmune disorder that predominantly affects young women, usually between 16 and 55 years of age [1–4]. It causes an innate and adaptive immune response leading to the appearance of autoreactive B and T lymphocytes and circulating autoantibodies [5]. It has a wide spectrum of medical manifestations [6–9]. These manifestations are usually related to autoantibodies, ensuing immune complex formation and deposition, and other immune processes [10]. Lupus patients can also experience the loss of self-tolerance [7]. They also have 2 to 10 times higher risk of suffering from coronary artery disease [11]. SLE has been previously considered as a rare disease, but over time it appears to be more common in certain population groups [12]. The age distribution of SLE cases is wide, however the incidence disease is usually highest at 15–44 years of age, while its prevalence is highest at 45–64 years of age [13]. The prevalence of SLE in the most recent study in Spain in the year 2000 was estimated to be about 91 cases per 100,000 inhabitants [14]. 

SLE is characterized by multiple organ damage outbreaks and affects more than five million people worldwide [15]. When this damage is found in the skin, it is classified as cutaneous lupus erythematosus (CLE) [2]. CLE occurs when there is chronic weakening of the skin, leaving significant skin damage such as scarring, atrophy and depigmentation [2]. In the past, there was a 10-year rate of survival that has currently improved to 92% thanks to the early recognition of milder cases and the improvement in general medical care [5, 11]. 

In 1971 the American Rheumatism Association created the first criteria to classify SLE, which have later been updated several times [16]. SLE is currently diagnosed according to the criteria established by American College of Rheumatology [2]. These criteria were last updated in 2017 with a new SLE classification system to support earlier diagnosis, with greater sensitivity and specificity [14]. The SLE treatment is based on the suppression of the immune response [1]. 

The objective of this study was to analyze the characteristics, use of health care resources and medical costs related to patients diagnosed with SLE and CLE in Spanish hospitals.

## Methods

### Study design

Inpatient admissions records were collected from a Spanish National discharge database [17] and analyzed in a retrospective multicenter study. The database covers 90% of hospitals in Spain from all regions. It is validated internally and subjected to periodic audits; during this span of time, errors and unreliable data are eliminated. All centers are responsible for the data codification, evaluation, and confidentiality.

An analysis of hospital admissions records of patients with CLE or SLE was performed. The database used collects data codified at the hospital level by means of the International Statistical Classification of Diseases and Related Health Problems, so the 10th version is used (ICD-10). The data inclusion period was from 1 January 2016 to 31 December 2020 with data from all Spanish regions.

### Data extraction

The ICD-10 codes used to identify patients were L93, corresponding to CLE patients and M32, corresponding to SLE patients. This research did not involve human participants and there was no access to identifying information, so in this case Spanish legislation does not require patient consent and ethics committee approval. There was no identification of healthcare centers or medical history, and to maintain anonymity in accordance with the principles of Good Clinical Practice and the Declaration of Helsinki, recoding was performed on all records.

### Study variables

The study variables analyzed included: patient’s age, national region, gender, type of admission, discharge type (including death), funding scheme, readmission, service, intensive care unit, length of hospital days, primary diagnosis, 19 secondary diagnoses, medical procedures, and total admission cost.

### Data analysis

Patients diagnosed with CLE or SLE were identified by the primary diagnosis code. They were classified by sex and into five age groups (below 18, between 18 and 44, between 44 and 65, between 66 and 85, and over 85). The first admission registered per patient was used to assess the characteristics of each patient, whereas all admissions files were used in the analysis of the admission details and the medical costs. Hospital incidence was measured as the ratio between admission and hospitalization rate per 10,000 persons based on the admissions registered in the database.

Direct medical costs were extracted from the database, where they are applied to each hospital admission according to the standardized average admissions costs and medical procedures identified by the Spanish Ministry of Health. These costs include all expenses regarding medical examinations, procedures, medications, surgery, diet, costs associated to personnel, medical equipment, and resources.

The Kolmogorov-Smirnov test [18] was used to test normality in all data. Frequencies and percentages are presented for dichotomous variables and mean or median were calculated for continuous variables. The Mann-Whitney U test [19] as a two-tailed non-parametric independent t-test or the Kruskal-Wallis test [20] as a one-way analysis of variance were used as appropriate and two-sample Z tests were used to differentiate in sample proportions. The Jonckheere-Terpstra trend test [21] was used to assess trends in incidence and cost. A *p* < 0.05 was considered to be statistically significant.

Microsoft Excel Professional Plus 2016 (Microsoft Corporation, Redmont, WA, USA), StataSE 12 for Windows (StataCorp LP. 2011. Stata Statistical Software: Release 12. College Station, TX, USA) and were used to perform statistical analysis.

## Results

During the study period, there were 416 admissions corresponding to 329 individual patients. Most cases were registered between the age range of 44 and 65 years old. The median age was 55 years of age and the 64.36% were females (Table 1). Most patients (93.60%) were diagnosed with SLE as a primary diagnosis, with a 26.75% diagnosed with unspecified SLE and a 25.53% with glomerular disease. From the total of patients diagnosed with CLE, a 65.22% had discoid CLE. The most common comorbidities were hypertension (31.61%), asthma (27.96%), hyperlipidemia (19.76%), nicotine dependence (17.02%), and chronic obstructive pulmonary disease (15.20%) (Table 2).

**Table 1:** Patient baseline characteristics

	Total
**Admissions, N**	416
**Patients, N**	329
0–17 years, N (%)	7 (2.13)
18–43 years, N (%)	85 (25.84)
44–65 years, N (%)	140 (42.55)
66–85 years, N (%)	91 (27.66)
>85 years, N (%)	6 (1.82)
**Average age**	54.65
2016 (%)	52.23
2017 (%)	58.63
2018 (%)	51.94
2019 (%)	54.76
2020 (%)	55.66
**Males, N (%)**	117 (35.56)
**Females, N (%)**	212 (64.44)

**Table 2:** Patient comorbidity profile

	Index admission	*p*-value^a^
**Admissions, N**	416	-
**Mean CCI**	2.75	< 0.0001
**Median CCI**	2	-
CCI 0, N (%)	1 (0.30)	< 0.0001
CCI 1, N (%)	29 (8.81)	< 0.0001
CCI 2, N (%)	177 (53.80)	< 0.0001
CCI 3, N (%)	45 (13.68)	< 0.0001
CCI 4, N (%)	36 (10.94)	< 0.0001
CCI 5, N (%)	23 (6.99)	< 0.0001
CCI > 5, N (%)	18 (5.47)	< 0.0001
**Mean updated CCI**	2.38	< 0.0001
**Median updated CCI**	2	-
CCI 0, N (%)	4 (1.22)	< 0.0001
CCI 1, N (%)	68 (20.67)	< 0.0001
CCI 2, N (%)	164 (49.84)	< 0.0001
CCI 3, N (%)	37 (11.25)	< 0.0001
CCI 4, N (%)	29 (8.81)	< 0.0001
CCI 5, N (%)	10 (3.04)	< 0.0001
CCI > 5, N (%)	17 (5.17)	< 0.0001
**Secondary diagnoses, > 10%**		
Essential (primary) hypertension, N (%)	104 (31.61)	< 0.0001
Unspecified asthma, without complications, N (%)	92 (27.96)	< 0.0001
Hyperlipidemia, N (%)	65 (19.76)	< 0.0001
Nicotine dependence, cigarettes, without complications, N (%)	56 (17.02)	< 0.0001
Chronic obstructive pulmonary disease, N (%)	50 (15.20)	< 0.0001
Personal history of nicotine dependence, N (%)	49 (14.89)	< 0.0001
Antiphospholipid syndrome, N (%)	40 (12.16)	< 0.0001
Old myocardial infarction, N (%)	39 (11.85)	< 0.0001
Long-term use of aspirin, N (%)	35 (10.63)	< 0.0001
**Mortality rate, %**	3.95	< 0.0001

^**a**^ index admission; CCI, Charlson’s Comorbidity Index

**Fig. 1:**
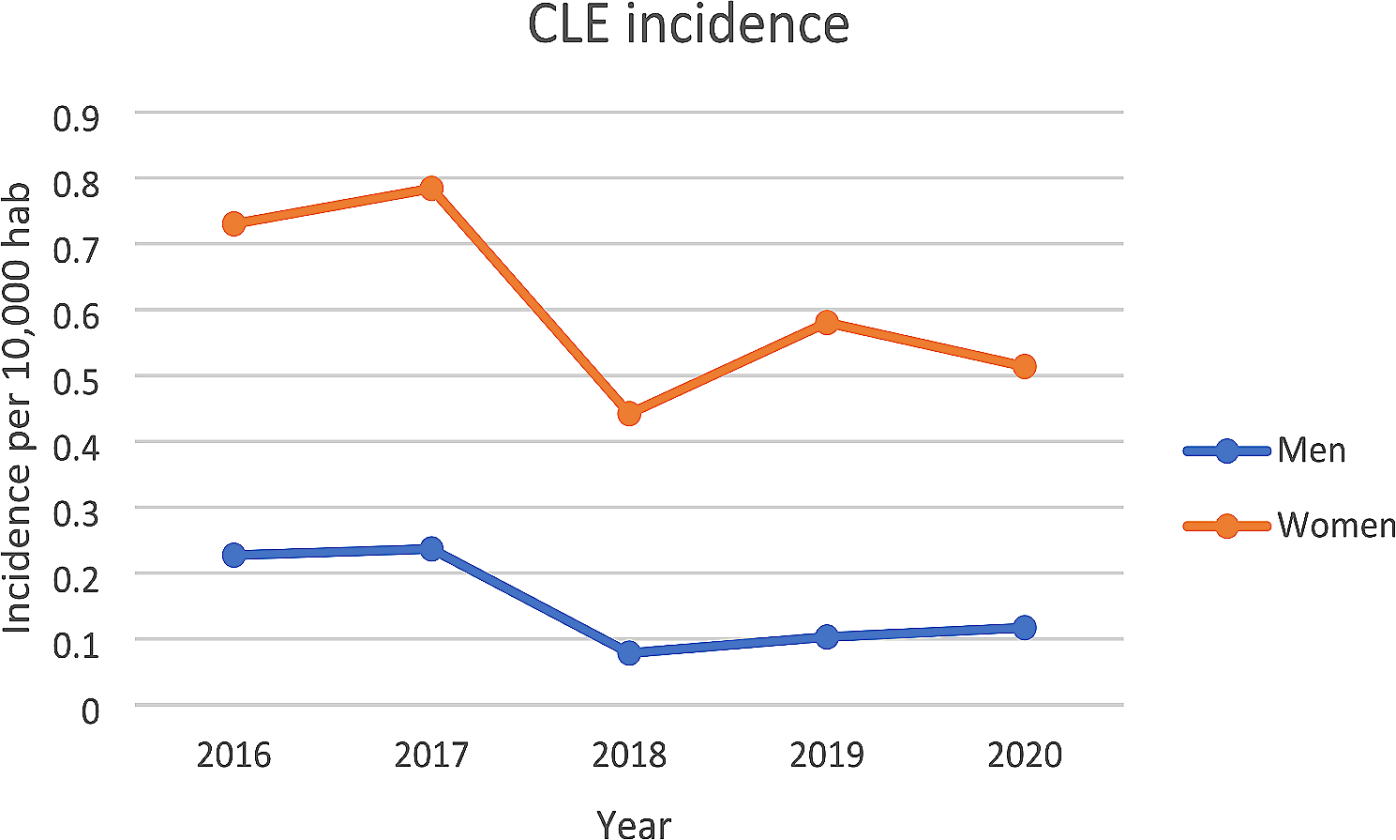
Incidence of CLE per 10,000 habitants

**Fig. 2:**
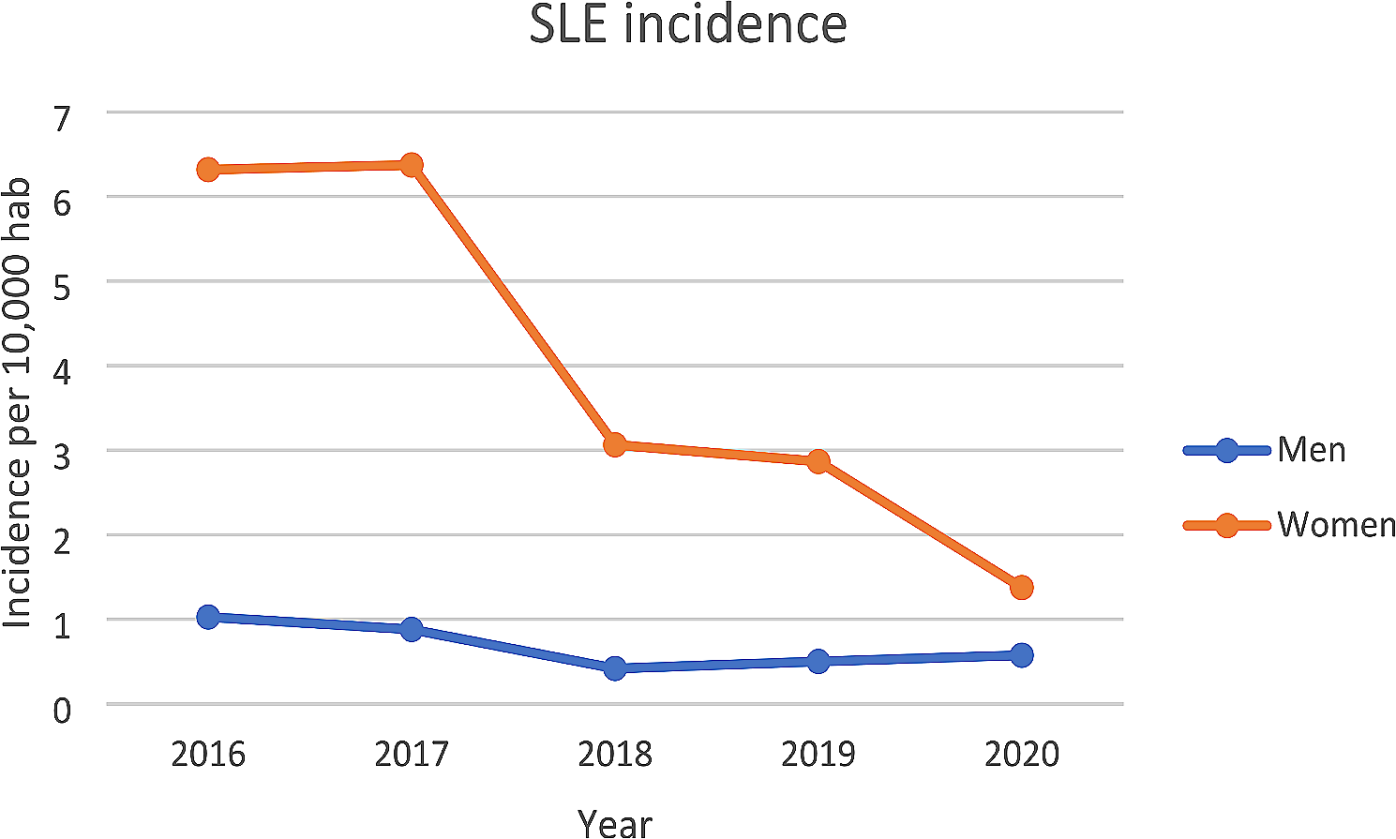
Incidence of SLE per 10,000 habitants

The incidence of CLE in hospitals was 0.49 per 10,000 persons in 2016 and 0.32 per 10,000 persons in 2020 (Fig. 1). It has decreased significantly between 2016 and 2020 (*p* = 0.0762 men, *p* = 0.0096 women) (Fig. 1). The incidence of SLE in hospitals was 3.67 per 10,000 persons and 1.76 per 10,000 persons in 2020 (Fig. 2). It has decreased significantly over the period between 2016 and 2020 (*p* = 0.0112 men, *p* < 0.0001 women) (Fig. 2).

The hospital department that received more admissions was the internal medicine department (39.42%), 21.39% were from nephrology and 20.91% were attended at the rheumatology department. The median length of hospital stays was 7 days. Of all the hospital admissions, 63.22% were urgent ones, while the rest were programmed or unknown. The most frequent medical procedures were introduction in peripheral vein of anti-inflammatory with percutaneous approach, plain chest x-ray, heart ultrasound, right and left sides, and abdominal ultrasound (Table 3).

**Table 3:** Medical procedures

	Index admission	*p*-value ^a^
**Medical procedures, > 10%**		
Heart ultrasound, right and left sides	64 (19.45)	< 0.0001
Introduction in peripheral vein of anti-inflammatory, percutaneous approach	59 (17.93)	< 0.0001
Plain chest x-ray	57 (17.33)	< 0.0001
Peripheral vein introduction of anti-infective, another anti-infective, percutaneous approach	40 (12.16)	< 0.0001
Abdominal ultrasound	39 (11.85)	
Measurement of electrical activity, cardiac, external approach	39 (11.85)	< 0.0001
Peripheral vein transfusion of red blood cells, non-autologous, percutaneous approach	34 (10.33)	< 0.0001
Kidney excision, right, diagnosis, percutaneous approach	33 (10.03)	< 0.0001

^**a**^ index admission

**Fig. 3:**
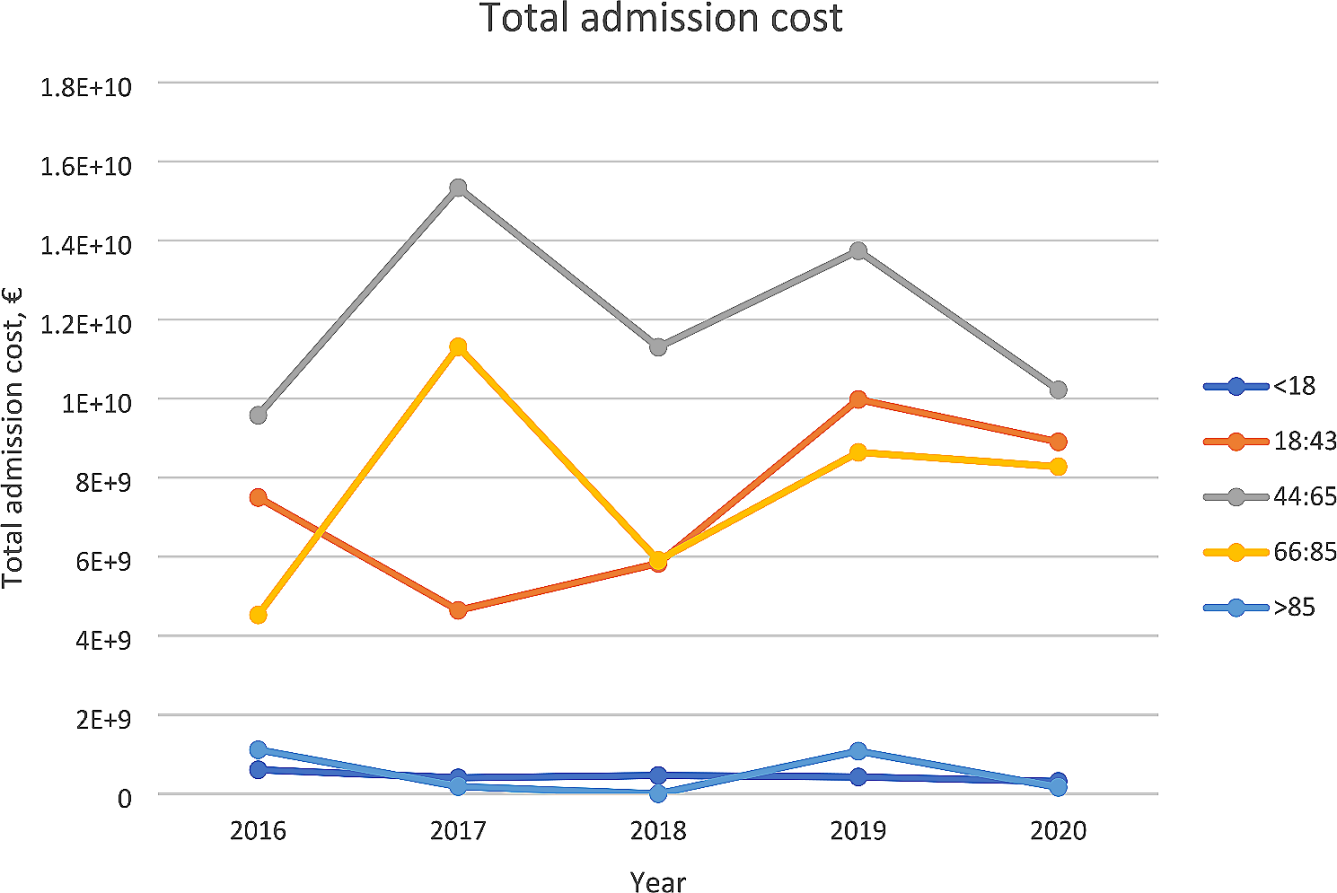
Total admission cost per group of age

The mean cost per each hospital admission over the study period was €6355.99. The total cost of lupus in Spain for the data inclusion period was €140,424.20 million, and €47,981.38 million for males, €92,442.83 million for females. The total annual cost had a statistical significance of *p* < 0.0001 to both, males and females (Fig. 3).

## Discussion

This retrospective study assessed the incidence and costs of lupus in Spain. This study found an incidence rate of lupus of 2.14 per 10,000 persons over the study period. There were significant differences between the incidence in men and women. The incidence in males was 0.60 per 10,000 persons, while in females it was 3.30 per 10,000. The age group with the highest incidence was between 15 and 44 years of age, but the study by Danchenko et al., (2006). showed a higher incidence found in the age group between 44 and 65 [13]. The average age of the patients in this study was 54.64 years of age. An article published in 2015 concludes that the patients with a higher age have a higher risk of future damage, particularly those who do not present with damage [22]. Traditionally SLE has been considered a disease affecting only young women, but recently, older populations from the age of 50 onwards have developed the disease [23]. In most studies, there is a female predominance in the patients being 90% of all the patients [3–7, 24]. An article published in 2017, showed that the sex ratio was 9:1 [25]. Meanwhile, an article published in 2011 showed that 1 out of 10 were male patients [6]. 

SLE has a relapsing-remitting pattern and usually develops over a prolonged period of time, so careful observation is required to make a diagnosis [1]. Since there is no sure cure for SLE, early diagnosis and treatment to manage dysfunction and complications is important [1]. Each patient, depending on the symptoms, will be treated individually to reduce the likelihood of permanent damage [1]. As a secondary condition, the respiratory system may be affected during the disease period [26]. The results show that 27.96% of the patients had asthma and 15.20% had chronic obstructive pulmonary disease. According to the results a 31.61% of the patients had hypertension as a secondary diagnosis, which can be correlated to the high risk of suffering from a coronary artery disease [11]. 

Regarding costs, lupus has shown to present high annual costs per hospital admission, with a mean of €6355.99. Similarly, the study of Fatoye et al. (2021) has shown mean annual costs of $7,740.19 for patients with SLE [27]. In addition, the estimation of the direct costs of SLE in this study showed significant variation compared to China (US$8,230), USA (US$13,305), the UK (€2,613) and Germany (€3,191) [28–30]. One of the reasons for the huge difference with the costs reported to the USA patients can be that the patients included in the USA’s study come from an US commercial insurance claims database and need to have at least 2 claims and a continuous health plan enrolment for six months to be included in the study. Also, the methods used to conduct the studies, including the differences in health care tariffs and the types of resources used in different countries, may be the major sources of variation when estimating direct costs of lupus patients.

## Conclusion

This study provides data describing the characteristics of patients who were diagnosed with lupus in Spanish hospitals over 5 years and the associated medical costs. The study showed a direct relation with gender, with more incidence and prevalence in the female population. A direct relationship between lupus and heart disease was also be detected. Total costs are expected to remain high in the coming years, although they seem to tend to stabilize in the same way as incidence. The trends observed in the hospital incidence must be further explored in the future to identify changes in the disease management over time.

## Data Availability

The datasets used and/or analyzed during the current study are available from the corresponding author on reasonable request. The data has been extracted from a database owned by the Ministry of Health at: https://www.sanidad.gob.es/estadEstudios/sanidadDatos/home.htm.

## Abbreviations

CCI: Charlson comorbidity index
CLE: Cutaneous lupus erythematosus
ICD-10: International Statistical Classification of Diseases and Related Health Problems 10th version
SLE: Systemic lupus erythematosus
